# P10-02 Barriers to use of the internet as an alternative delivery channel for an evidence-based fall-prevention intervention for older adults

**DOI:** 10.1093/eurpub/ckac095.141

**Published:** 2022-08-29

**Authors:** Dina Jones, Maura Robinson, Terry Kit Selfe, Lucinda Barnes, Sijin Wen, Samantha Shawley-Brzoska, Douglas Myers, Sara Wilcox

**Affiliations:** 1 Department of Orthopaedics & Physical Therapy, West Virginia University, Morgantown, USA; 2 Health Science Center Libraries, University of Florida, Gainesville, USA; 3 Mountaineer Doctor Television, West Virginia University, Morgantown, USA; 4 Department of Biostatistics, West Virginia University, Morgantown, USA; 5 Office of Health Services Research, West Virginia University, Morgantown, USA; 6 Department of Community and Environmental Health, Boise State University, Boise, USA; 7 Arnold School of Public Health & Exercise Science, University of South Carolina, Columbia, USA

**Keywords:** exercise, fall prevention, internet, intervention, older adults

## Abstract

**Issue/problem:**

Physical activity (PA) can prevent falls, a leading cause of death globally. Alternative delivery channels may increase the “reach” of interventions into older adult populations in areas which lack trained instructors. Using technology is one alternative to traditional, face-to-face group classes where instructors and participants are in the same room. We delivered a PA program via the Internet for older adults in rural West Virginia (USA). This alternative could help other countries reach more older adults and reduce falls.

**Description of the problem:**

Tai Ji Quan: Moving for Better Balance® (TJQMBB) is an evidence-based intervention for older adults that reduces falls. Adults, ≥ 55 years, attended free, 1-hour tele-TJQMBB sessions, twice weekly, for 16 weeks at 5 remote community sites (3 urban, 2 rural). Trained instructors (new to TJQMBB) led 6 classes from a classroom for participants at remote sites. Instructors/sites used minicomputer, web camera, microphone, and television(s) for live, 2-way verbal/audio exchange. A CPR-certified person was present with participants. This project identified barriers to implementing tele-TJQMBB. Data on barriers were collected from instructors' class logs and summarized.

**Results:**

Fifty-two adults (81% female, mean age 70) attended 23 (median) of 32 sessions. Barriers that caused session cancelations included: participant vacations/unavailability (n = 7), inclement weather (n = 4), technical issues (n = 2), no CPR person (n = 1), classroom not available (n = 1), site closed for state holiday (n = 1), competing event at site (n = 1), and ill instructor (n = 1). Technical barriers during sessions included interruptions/lack of audio, video freezing, and Wi-Fi/Internet connectivity problems. Two participants did not like videoconferencing.

**Lessons:**

Tele-TJQMBB may be easier to teach with instructors who have already taught the program in-person. Selecting instructors/sites that are comfortable with technology may reduce technological barriers. Some older adults may still prefer face-to-face classes. Most cancelations were due to reasons normally encountered in face-to-face classes. Use of technology added new barriers that will need addressed for future classes.

**Main messages:**

Technology reached older adults in areas with no instructors. We think that this is the first time a live, evidence-based, group PA intervention was delivered using this method which could be a model for reaching older adults globally.

